# Experimental Study on Bond-Slip Behavior between Corroded I-Shaped Steel and Concrete in Subsea Tunnel

**DOI:** 10.3390/ma12182863

**Published:** 2019-09-05

**Authors:** Mingnian Wang, Yiteng Zhang, Li Yu, Yucang Dong, Yuan Tian, Guojun Zhou

**Affiliations:** 1 Key Laboratory of Transportation Tunnel Engineering, Ministry of Education, Southwest Jiaotong University, Chengdu 610031, China; 2 School of Civil Engineering, Southwest Jiaotong University, Chengdu 610031, China; 3 China Railway Eryuan Engineering Group Co. LTD, Chengdu 610031, China

**Keywords:** steel reinforced concrete, corrosion, push-out test, bond–slip behavior, bond stress, degradation constitutive model

## Abstract

Degradation of the bond between I-shaped steel and concrete due to the corrosion of I-shaped steel significantly affects the durability of steel reinforced concrete (SRC) structures. This study carried out the accelerated corrosion test and push-out test to study the bond-slip behavior and characteristics considering the corrosion of I-shaped steel, and test results indicated that: (1) The performance degradation of the bond-slip accelerated when the corrosion ratio reached 12%. (2) The corrosion failure pattern of SRC experienced slip phase and destruction phase in the rising stage. (3) Based on the principle of minimum potential energy, the bond stress was obtained only with the load and the displacement in the free end and the loading end. (4) Meanwhile, a new bond-slip degradation model was developed using the interface damage theory. Finally, the proposed model agreed with the experimental results.

## 1. Introduction

The subsea tunnels are often constructed by drilling and blasting method. For example, the subsea tunnels in Norway were all constructed by drilling and blasting method [1], and the Japanese Seikan Tunnel (53.8 km) was also constructed by drilling and blasting method [2]. In China, the drilling and blasting method was used in Xiang’an Submarine Tunnel (5.9 km) in Xiamen [3], and Jiaozhou Bay Submarine Tunnel (6.17 km) in Qingdao [4]. The composite lining composed of initial support and secondary lining is often used in the subsea tunnel support system constructed by drilling and blasting method. Since SRC has several advantages over the traditional reinforced concrete (RC): these include high bearing capacity, high rigidity, good ductility and energy dissipation [5,6], I-shaped steel is generally installed in the initial support in the soft rock section of the subsea tunnel. Shotcrete employed for initial support is sprayed onto the substrate at a high speed through a pneumatic hose or pipe under the action of air pressure and compacted instantaneously [7]. Compared to ordinary shotcrete, shotcrete has better Cl^−^ penetration properties, and Wang et al. [8] attributed these to the porosity generated during shooting and the “looseness” of the microstructure due to the fast formation of ettringite and C-S-H gel. Therefore, the I-shaped steel corrosion in the initial support of subsea tunnels occurred easily due to the electrochemical action caused by chloride ions invasion. When the cumulative concentration of chloride ions reaches a certain level, the passive film of steel will be activated and the electrochemical reaction will happen [9,10]. The electrochemical reaction of I-shaped steel can be presented as: *Fe → Fe^2+^*+2*e*^−^ at the anode, and O_2_ + 2H_2_O + 4*e*^−^ → 4*OH*^−^ at the cathode [11,12]. With the I-shaped steel gradually corrode, and the bond strength between the I-shaped steel and shotcrete will gradually weaken, which changes the mechanical properties of the initial support and has a negative impact on the safety of the whole initial support system. Therefore, it is of great significance to study the bond-slip law between corroded I-shaped steel and shotcrete in initial support, especially the bond-slip constitutive model relationship for evaluating the performance of initial support.

The study on the corrosion effect of seawater on reinforced concrete structures in ocean engineering is highly valued, and its durability is clearly stipulated in the codes of various countries in the world. At the same time, there are many studies on bond-slip behavior of corroded reinforced concrete. Abundant achievements have been made in the following three aspects: the law of bond strength degradation caused by reinforcement corrosion, the average bond stress-slip relationship of corroded reinforced concrete, and the bond-slip constitutive relationship of corroded reinforced concrete considering the influence of anchorage position [13,14,15,16,17,18,19,20,21,22]. However, for the subsea tunnels, I-shaped steel concrete is different from ordinary reinforced concrete in the following four aspects: (1) The contact area between I-shaped steel and concrete is larger (the difference can be five times); (2) The spatial form of I-shaped steel and steel bar is different, which leads to the difference of mechanical properties; (3) The corrosion of I-shaped steel is uneven, and corrosion in the flange side is usually bigger than that in the web side; (4) There is uneven expansion force between corroded I-shaped steel and concrete. Therefore, the bond-slip law after corrosion may be quite different.

At present, there is some research on the bond behavior and characteristics of steel reinforced concrete (SRC) structures. Bryson et al. [23] performed the first push-out test to studied the effect of section steel surface condition on bond strength of steel reinforced concrete, the results showed that the average bond strength of section steel either freshly sandblasted or sandblasted and allowed to rust was close, but it is about 30% higher than that of section steel with normal rust and mill scale. However, at a free-end slip of 0.0254 mm, there was no significant difference in the bond stress for all three types of surface conditions. Roeder [24] found that the bond strength distribution along the anchorage length was similar to the exponential distribution, and the bond strength between the reinforced concrete and an embedded shape steel was sufficient to bear the ultimate load without shear connectors. Hamdan et al. [25] performed the push-out test to focus on the factors influencing steel reinforced concrete bond strength, including concrete strength, surface condition of section steel and transverse stirrup ratio. The test results showed that the concrete strength had no obvious influence on the bond strength of steel reinforced concrete. Increasing the transverse stirrup ratio and sand blasting treatment on the surface of steel could improve the bond strength of steel reinforced concrete. Yang et al. [26] conducted a series of SRC push-out test, defined three key bond strength, three key slip and proposed a mathematical model of bond-slip constitutive relationship according to the load-slip curves. Zheng et al. [27] analyzed the bond-slip mechanism of steel and concrete, the main factors affecting the bond-slip performance and the distribution mode of bond stress and slip along the embedment length, established the formulas for calculating the bond strength and slip, put forward the calculating principle and method for the ultimate load of local bond failure and overall bond failure. Zheng et al. [28] carried out the push-out test of ten partially concrete encased steel specimens; the experiments have found that the concrete strength grade, contact surface state and contact length had great influence on the bond-slip performance. Liu et al. [29] carried out twenty-seven standard push-out specimens to study bond-slip behavior between section steel and RAC (Recycled Aggregate Concrete). Studies have shown that RCA, RAC strength, embedment length as well as thickness of concrete cover of SRC have influence on average bond strength of SRRC (Steel Reinforced Recycled Concrete). Chen et al. [30] carried out eleven push-out specimens experiments based on the orthogonal design method to investigate the effects of a checkered steel plate pattern height, position of pattern and transverse stirrup ratio on bond strength. The experiment results revealed that the checkered pattern steel plates placed on between the shaped steel (reinforcement area) and the concrete could significantly improve bond strength. Wang et al. [31] conducted the steel-concrete specimens test at a high temperature. The results showed that the ultimate slip load and residual load of the shaped steel concrete were significantly lower than those at room temperature, and the decline was fastest in the range from 20 to 200 °C.

In this investigation, with respect the effects of corrosion ratio of I-shaped steel, this paper adopted accelerated corrosion test and push-out test to obtain bond-slip behavior and bond-slip degradation constitutive model between corroded I-shaped steel and concrete. It not only has practical application value in guiding the design, construction and maintenance of support system of subsea tunnel, but also enriches and develops the bond-slip degradation theory of corroded steel reinforced concrete structure.

## 2. Experimental Program

### 2.1. Accelerated Corrosion Test

It is well known that natural chloride corrosion of I-shaped steel embedded in concrete takes up to several years or more, which makes it difficult to carry out in a laboratory. Thus, the corrosion of I-shaped steel is accelerated by accelerated corrosion test. According to Faraday’s law, as expressed by Equation (1).

(1) $$\Delta m=\frac{tMiS}{zF}$$

where *t* is the estimated corrosion age (s), *F* is Faraday’s constant (96,500 C/mol), *z* is the ionic charge number (z = 2) of iron, △*m* is the target mass loss of corroded reinforcement (g), *M* is the molar mass of iron (56 g/mol), *i* is the corrosion current density (A/cm^2^), and *S* is the superficial area of I-shaped steel within the corrosion region (cm^2^).

Although the corrosion mass can be directly obtained from Equation (1), the electrolytic corrosion test is affected by a variety of interference factors, and the theory is quite different from the practice. Therefore, this paper determines the quantitative relationship between the corrosion mass (△*m*) and the corrosion time (*t*) through the accelerated corrosion test of I-shaped steel concrete with five small samples.

#### 2.1.1. Preparation for Testing

**Figure 1:**
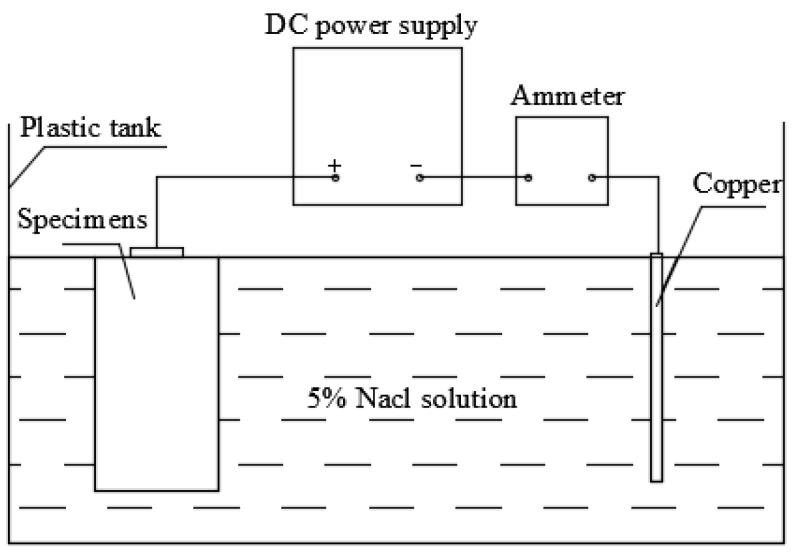
Electrolytic cell.

The main device used in accelerated corrosion test was the electrolytic cell composed of direct-current power supply, ammeter, wire, 5% NaCl solution, copper sheet, as shown in Figure 1.

**Figure 2:**
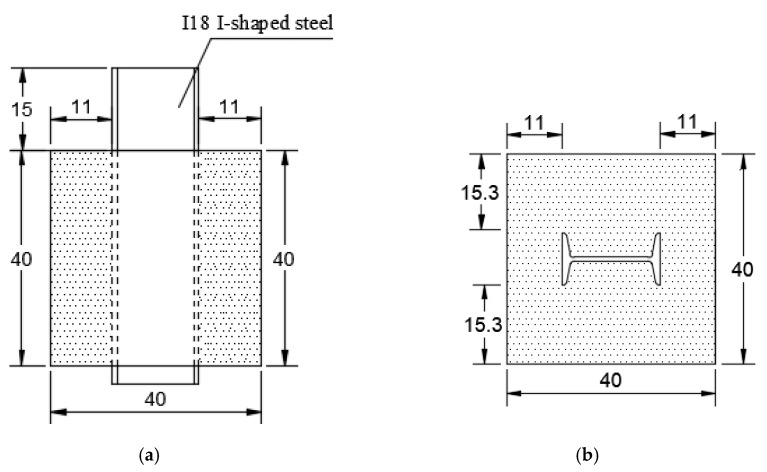
Schematic diagram of specimens (unit: cm). (**a**) Longitudinal section; (**b**) Cross section.

According to the actual design of Xiang’an Subsea Tunnel, the specimens adopted NO.18 I-shaped steel with equal length. The mass proportion of concrete was water:gravel:sand:cement = 1:3.5:2.7:1.8. The type of concrete was C25. The parameters of I-shaped steel and concrete were shown in Table 1. The specific size of the specimens was shown in Figure 2, the cross section was a square section of 40 cm × 40 cm, I-shaped steel was arranged symmetrically along the central axis of the cross section.

#### 2.1.2. Testing Procedure

**Figure 3:**
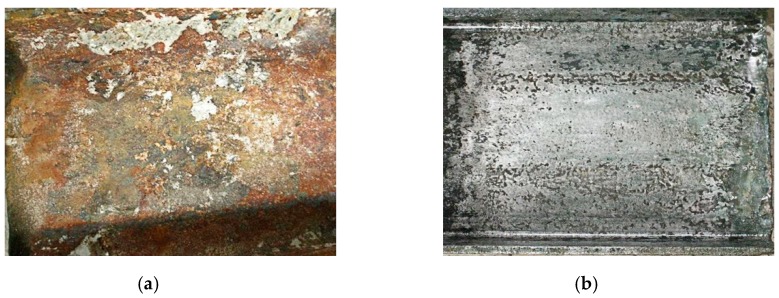
The state of I-shaped steel. (**a**) I-shaped steel after corrosion; (**b**) I-shaped steel after corrosion rust removal.

The accelerated corrosion test was carried out in the following steps:

Step 1: 5% NaCl solution was configured in the electrolytic cell. The specimens were placed into the electrolytic cell and soaked for two days to make the salt water infiltrate into the specimens and contact with the I-shaped steel inside;

Step 2: Connecting the electrolytic circuit, as shown in Figure 1. Note that direct-current power supply was used here;

Step 3: Opening the power supply and adjusting the current, the control current was 5A;

Step 4: Maintain the current constant, electrify to the original design time, take out the components, chisel out I-shaped steel (seen in Figure 3a);

Step 5: The corrosion mass of each specimen was determined by measuring the weight of the I-shaped steel after removing the rust (seen in Figure 3b) with 12% hydrochloric acid solution for pickling and with 3% sodium carbonate solution neutralization.

#### 2.1.3. The Relationship between the Mass Loss and Conducting Time

According to accelerated corrosion test, Table 2 could be obtained.

**Figure 4:**
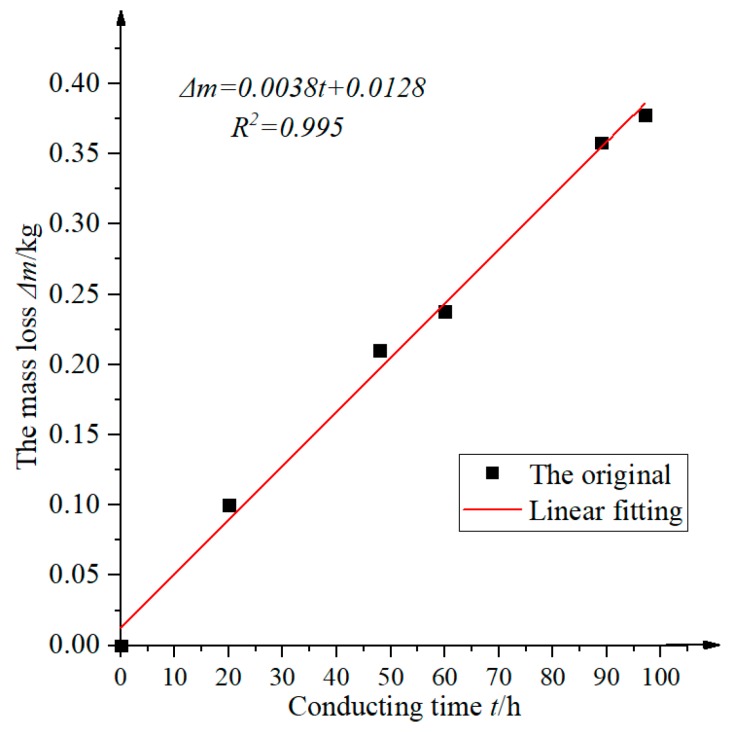
The relationship between the mass loss and conducting time.

Table 2 was represented by curve, as shown in Figure 4.

It can be seen from Figure 4, under constant current (5A), the relationship between the mass loss and the corrosion time was linear, and the test results were consistent with Faraday’s law. Therefore, we obtained the relationship between the mass loss and the conducting time in our accelerated corrosion tests, as expressed by Equation (2).

(2) Δm=0.0038t+0.0128

where △*m* is the mass loss of corroded I-shaped steel (kg), *t* is the conduction time (h). Note that the premise of Equation (2) is that the control current is 5A. According to Equation (2), the corrosion mass can be obtained from the corrosion time, and then the corrosion ratio ρ can be obtained by dividing the total mass (Equation (3)), which lays a foundation for the subsequent bond-slip degradation test.

(3) $$\rho =\frac{\Delta m}{m}\times 100\%$$

### 2.2. Push-Out Test

#### 2.2.1. Testing Method and Specimen Preparation

The specimens produced in the test were the same as those produced in accelerated corrosion test. There were 6 groups in this test, one group of I-shaped steel was not corroded, the other 5 groups were corroded (all the current was 5A), and the mass loss was obtained from the conducting time according to Equation (2), and then the corrosion ratio was obtained by Equation (3), as shown in Table 3.

#### 2.2.2. Setup for Push-Out Test

**Figure 5:**
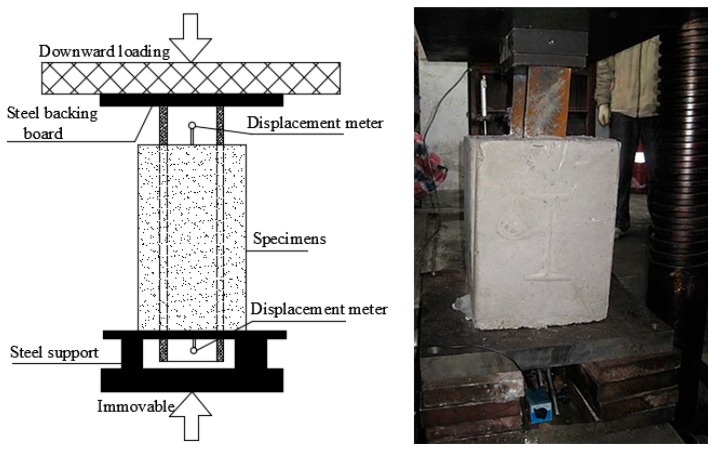
Loading device.

The push-out test was conducted using a custom-built loading setup with pressure testing machine with a maximum capacity of 5000 kN, as shown in Figure 5. All the specimens were loaded under loading speed control with a constant loading rate of 40 kN/min, per 20 kN was one level in the loading process, until the bond was broken. The lower end of the concrete was fixed on the test bench by the support and the upper end was free. When loading, the steel back board pushed down the I-shaped steel, and the lower end of the concrete bear the load from the pressure testing machine. Therefore, the upper end of the I-shaped steel was the loading end and the lower end was the free end. Two displacement meters with accuracy of 0.01 mm were used to monitor the slip displacement at both the free end and loading end of the specimens.

## 3. Test results and Discussion

### 3.1. Fracture Failure Process

**Figure 6:**
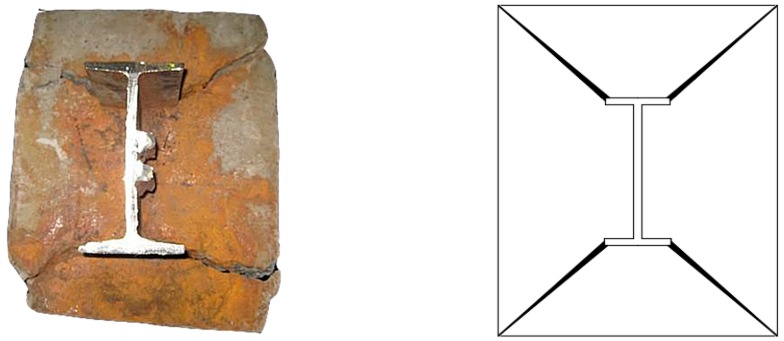
Typical crack patterns at loading ends.

At the initial stage of loading, the displacement sensors at the loading and free ends were basically unchanged due to the smaller load, which meant that there was almost no relative slip generated inside. As the applied load continued to increase, the slip at the loading end increased slowly and the cracks at the end face developed rapidly. The cracks began to appear from the two ends of the flange, and rapidly expanded from the inside to the outside with the increase of the load, as shown in Figure 6. When the applied load reached or just exceeded the bond strength of the specimens, the load readings of the pressure testing machine had fallen back rapidly; the pressure testing machine could not continue loading.

### 3.2. The Relationship between Bond Failure Load and Corrosion Ratio

**Figure 7:**
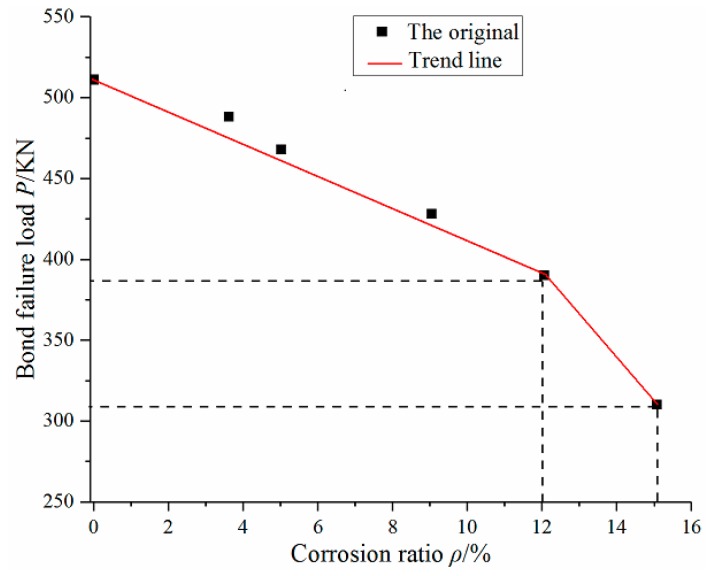
Relationship between corrosion ratio and bond failure load.

Through the six groups of push-out test, the scatter plot of corrosion ratio and bond failure load can be obtained, as shown in Figure 7.

Through the observation and analysis of Figure 7, with the increase of the corrosion ratio, the bond failure load of the specimens presents a decreasing trend. When the corrosion ratio is less than 12%, the trend decreases linearly. When the corrosion ratio is greater than 12%, the decline rate increases suddenly. Therefore, the corrosion ratio of 12% is a turning point for accelerating degradation of bond-slip performance.

### 3.3. Load-Slip Curve

**Figure 8:**
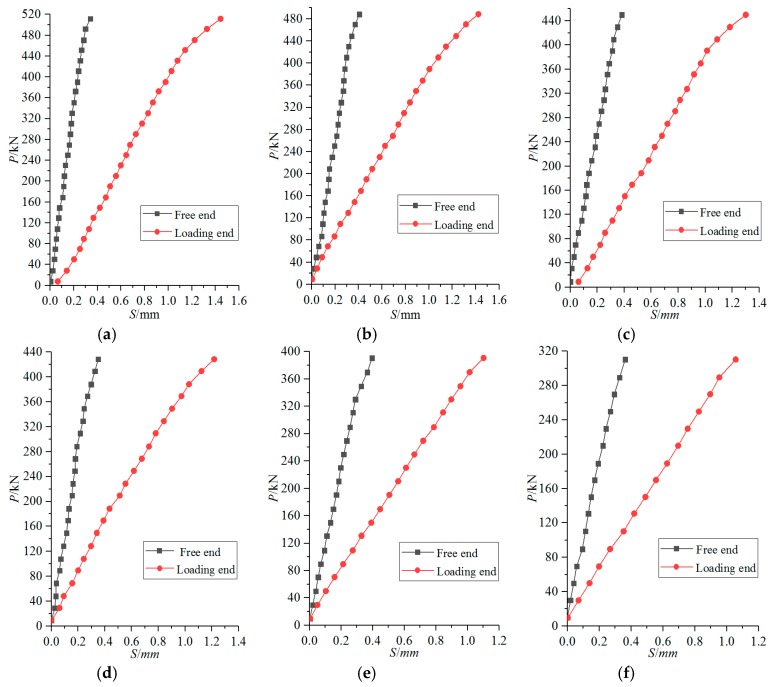
Actual loading end and free end P-S curves of specimens. (**a**) SRC-1; (**b**) SRC-2; (**c**) SRC-3; (**d**) SRC-4; (**e**) SRC-5; (**f**) SRC-6.

Figure 8 is the load slip (*P-S*) curve of the loading end and the free end of the specimens. Due to the limitations of experimental conditions, only the rising segment of the load-slip (*P-S*) curve is obtained in this experiment.

Through the observation and analysis of Figure 8, the *P-S* curve of the corroded steel reinforced concrete in the rising segment can be roughly divided into the following two stages:

(1) Slip phase: Once loading begins, the loading end and free end also begin to slip and develop stably. However, the slip value at the loading end is significantly greater than that at the free end. In this stage, the slip value of the loading end is about 0.04–0.07mm per 20 kN, while that of the free end is about 0.01–0.03 mm. The curve at this stage is approximately linear until the load reaches about 85% of the ultimate load. 

(2) Destruction phase: When the load is close to 85% of the ultimate load, the sliding development of the loading end and the free end is accelerated. At this time, the slip value of the loading end is about 0.07–0.12 mm per 20 kN, while that of the free end is about 0.02–0.04 mm. When the applied load is close to the ultimate load, longitudinal cracks will suddenly appear at the loading end, or the existing cracks caused by the original corrosion will develop rapidly and widen. When the load reaches the ultimate load, bond failure occurs.

### 3.4. Distribution of Bond Stress along Specimen Length

In the push-out test, the internal bond stress test was not carried out due to corrosion, but the distribution law of bond stress along anchorage length was obtained by analyzing the push-out test based on the energy method.

**Figure 9:**
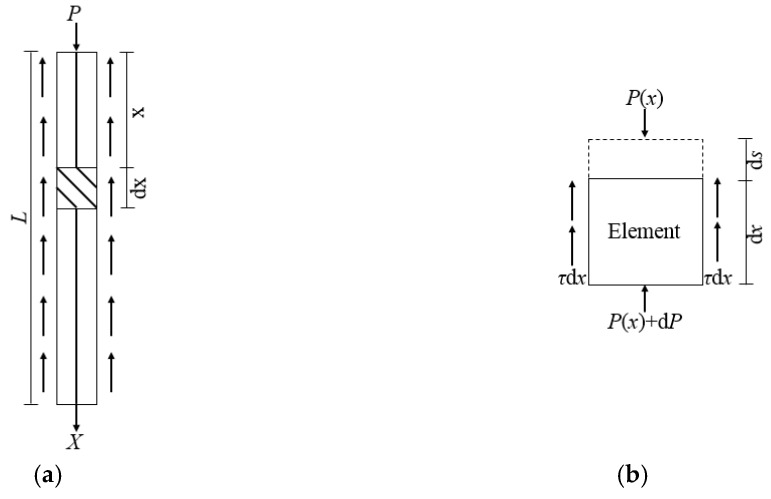
Diagram of force analysis of I-shaped steel. (**a**) Force diagram of detached body; (**b**) Force diagram of element body.

In order to analyze the relative slip between I-shaped steel and concrete, I-shaped steel is taken as the detached body, and its stress is shown in Figure 9a. Take any I-shaped steel element from the detached body, and its stress is shown in Figure 9b.

It is assumed that the bond stress of the I-shaped steel is the same everywhere at the same anchorage depth. Equation (4) can be obtained from the static equilibrium condition [32] of I-shaped steel.

(4) $$\tau \left(x\right)C_{s}d_{x}+dP_{s}\left(x\right)=0$$

where *τ(x)* is the bond stress between I-shaped steel and concrete at *x*, *P_s_(x)* is the axial force of I-shaped steel at *x*, *C_s_* is the perimeter of I-shaped steel section.

It is assumed that the longitudinal deformation of I-shaped steel at x is *S_s_*(*x*), and the relationship between axial force, elastic deformation and longitudinal strain of concrete is obtained by Equation (5), according to Hooke’s law [32].

(5) $$P_{s}\left(x\right)=-E_{s}A_{s}\frac{dS_{s}\left(x\right)}{d_{x}}=-E_{s}A_{s}\varepsilon_{s}\left(x\right)=-A_{s}\sigma_{s}\left(x\right)$$

where *ε_s_*(*x*) is axial strain of I-shaped steel at *x*, *σ_s_*(*x*) is axial stress of I-shaped steel at *x*, *E_s_* is elastic modulus of I-shaped steel, *A_s_* is cross-sectional area of I-shaped steel.

Substitute Equation (5) into Equation (4) to get Equation (6).

(6) $$\tau \left(x\right)C_{s}-E_{s}A_{s}\frac{d^{2}S_{s}\left(x\right)}{dx^{2}}=0$$

Similarly, the concrete element at x is taken as the element body, and Equation (7) is obtained.

(7) $$\tau \left(x\right)C_{s}+E_{c}A_{c}\frac{d^{2}S_{c}\left(x\right)}{dx^{2}}=0$$

where *S_c_*(*x*) is the longitudinal deformation of concrete at x, *E_c_* is elastic modulus of concrete, *A_c_* is cross-sectional area of concrete.

In the state of use, the deformation of I-shaped steel and concrete can be regarded as elastic, so the relative slip of them at x can be obtained by their displacement difference, as expressed by Equation (8).

(8) $$S\left(x\right)=S_{s}(x)-S_{c}(x)$$

According to the test results in reference [24,33], it is deduced that the bond stress of steel reinforced concrete presents an exponential distribution along the anchorage length, which can be expressed by Equation (9).

(9) $$\tau \left(x\right)=Ae^{Bx}$$

where parameters A and B are undetermined coefficients.

Substitute Equation (9) into Equations (6) and (7) to obtain two second-order constant coefficient differential equations, when *K_s_* and *K_c_* are respectively represented by Equations (10) and (11), the general solution expression is expressed by Equation (12).

(10) $$K_{s}=\frac{AC_{s}}{E_{s}A_{s}}$$

(11) $$K_{c}=-\frac{AC_{s}}{E_{c}A_{c}}$$

(12) $$\{\begin{array}{l} S_{s}\left(x\right)=C_{1}+C_{2}x+\frac{K_{s}}{B^{2}}e^{Bx}\\ S_{c}\left(x\right)=C_{3}+C_{4}x+\frac{K_{c}}{B^{2}}e^{Bx} \end{array}$$

where *C_1_*, *C_2_*, *C_3_* and *C_4_* are undetermined coefficients.

Substitute Equation (12) into Equation (8), the expression of relative slip between I-shaped steel and concrete can be obtained, as expressed by Equation (13).

(13) $$S\left(x\right)=C_{1}-C_{3}+\left(C_{2}-C_{4}\right)x+\left(K_{s}-K_{c}\right)\frac{1}{B^{2}}e^{Bx}$$

In the push-out test of steel reinforced concrete, the slip value *S*(0) at the loading end can be measured by setting a displacement meter at the loading end. Therefore, the difference between coefficient *C_1_* and *C_3_* in Equation (13) can be calculated by the slip test value at the loading end, as expressed by Equation (14).

(14) $$C_{1}-C_{3}=S\left(0\right)-\left(K_{s}-K_{c}\right)\frac{1}{B^{2}}$$

For linear elastic materials, the necessary and sufficient condition for allowable displacement *S*(*x*) to be true displacement is that the total potential energy ∏ of the system is minimum [34] according to the principle of minimum potential energy, simultaneously satisfying Equation (15).

(15) $$\frac{\partial \prod }{\partial C_{i}}=0$$

where *C_i_* is the unknown quantity in Equation (12), *i* = 2,4.

The total potential energy of the system consists of two components [34], as expressed by Equation (16).

(16) ∏=U+W

where *U* is the strain energy of the whole structure, *W* is the external potential energy of the whole structure.

For the integral members, the strain energy of I-shaped steel and concrete is expressed by Equation (17).

(17) $$\{\begin{array}{l} U_{\varepsilon s}=\frac{E_{s}A_{s}}{2}{\int_{0}^{l}\left(\frac{dS_{S}}{dx}\right)}^{2}dx\\ U_{\varepsilon c}=\frac{E_{c}A_{c}}{2}{\int_{0}^{l}\left(\frac{dS_{c}}{dx}\right)}^{2}dx \end{array}$$

The external potential energy is expressed by Equation (18).

(18) $$W=-\int_{0}^{l}\tau \left(x\right)C_{s}S\left(x\right)dx-PS_{s}\left(0\right)$$

Substitute Equations (17) and (18) into Equation (16) to get the total potential energy of the system, as expressed by Equation (19).

(19) $$\prod =\frac{E_{s}A_{s}}{2}{\int_{0}^{l}\left(\frac{dS_{S}}{dx}\right)}^{2}dx+\frac{E_{c}A_{c}}{2}{\int_{0}^{l}\left(\frac{dS_{c}}{dx}\right)}^{2}dx-\int_{0}^{l}\tau \left(x\right)C_{s}S\left(x\right)dx-PS_{s}\left(0\right)$$

According to Equation (15), *C_2_* and *C_4_* can be obtained, as expressed by Equation (20).

(20) $$\{\begin{array}{l} C_{2}=\frac{K_{s}}{B^{2}l}\left(Ble^{Bl}-2e^{Bl}+2\right)\\ C_{4}=\frac{K_{c}}{B^{2}l}\left(Ble^{Bl}-2e^{Bl}+2\right) \end{array}$$

Therefore, under the action of external load *P*, the relative slip at any section of the steel reinforced concrete can be obtained, as expressed by Equation (21).

(21) $$S\left(x\right)=S\left(0\right)+\left(K_{c}-K_{s}\right)\frac{1}{B^{2}}+\left[\frac{1}{B^{2}l}\left(K_{s}-K_{c}\right)\left(Ble^{Bl}-2e^{Bl}+2\right)\right]x+\left(K_{s}-K_{c}\right)\frac{1}{B^{2}}e^{Bx}$$

By substituting the anchorage length *l* of I-shaped steel into Equation (21), the relational expressions of slip *S*(*0*) at loading end, slip *S*(*l*) at free end and bond stress coefficient *A* and *B* can be obtained, as expressed by Equation (22).

(22) $$S\left(l\right)=S\left(0\right)+\frac{\left(K_{s}-K_{c}\right)}{B^{2}}\left(Ble^{Bl}-e^{Bl}+1\right)$$

Based on balance condition of forces [32], Equation (23) can be obtained.

(23) $$\int_{0}^{l}\tau \left(x\right)C_{s}dx=P$$

According to Equation (23), the relationship between external load P and coefficients A and B can be obtained, as expressed by Equation (24).

(24) $$e^{Bl}-1=\frac{PB}{C_{s}A}$$

The relative slip *S*(0) of the loading end and *S*(*l*) of the free end are measured by setting a displacement meter at both ends of the I-shaped steel (Figure 5). Based on the above analysis, under the premise that the geometrical and physical parameters of the specimen are certain, the concrete parameters *A* and *B* of the bond stress distribution curve can be obtained by Equations (22) and (24), which only need the corresponding information of the external load *P* and the slip *S*(0) and *S*(*l*) at both ends of the specimens.

Since the energy analysis method adopted in this paper is based on the linear elastic theory, Equation (21) is only applicable to the linear elastic stage of bond-slip relationship. From the research results [35], it can be seen that in the bond-slip curve of steel reinforced concrete, before the applied load reaches 80% of the bond failure load, the bond stress increases linearly with the slip, that is, the bond-slip relationship is in the linear elastic stage. Therefore, it can be considered that Equation (21) is suitable for calculating the local slip of steel reinforced concrete with a push-out load less than 80% of the bond failure load.

**Figure 10:**
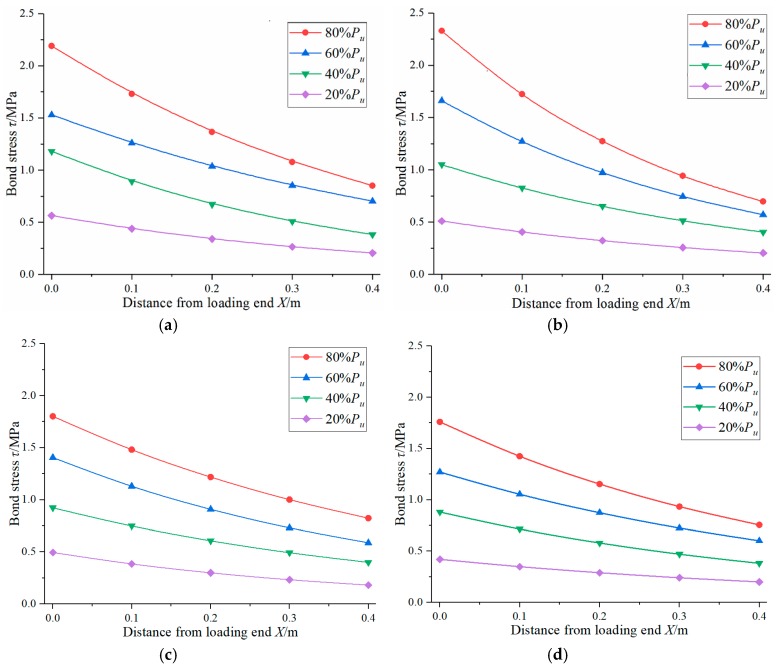

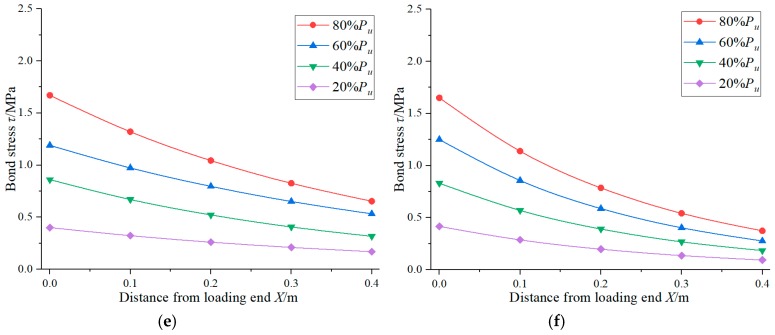
The distribution of bond stress based on theoretical analysis. (**a**) SRC-1; (**b**) SRC-2; (**c**) SRC-3; (**d**) SRC-4; (**e**) SRC-5; (**f**) SRC-6.

According to the measured *P-S* curve in the push-out test of steel reinforced concrete and the parameters of the specimens (Table 4), the distribution of bond stress calculated by the Equations (22) and (24) in this paper is shown in Figure 10.

### 3.5. Study on Interface Damage of Corroded I-Shaped Steel and Concrete

In the study of damage mechanics, it is necessary to select reasonable variables to describe the damage state of materials and structures. The interface damage between I-shaped steel and concrete due to corrosion cannot be obtained by simple test means, so the area of interface damage cannot be simply used to determine the damage variable. Wu et al. [36,37] used the bond strength of the interface to define the damage variable, as expressed by Equation (25).

(25) $$D=\frac{\left(P_{u}/A-\bar{P_{u}}/A\right)}{P_{u}/A}$$

where *P_u_* is the limit load of I-shaped steel concrete before interface damage, $\overset{-}{P_{u}}$ is the limit load of I-shaped steel concrete after interface damage, *A* is the surface area of I-shaped steel wrapped by concrete, here is A = 289,200 mm^2^.

**Figure 11:**
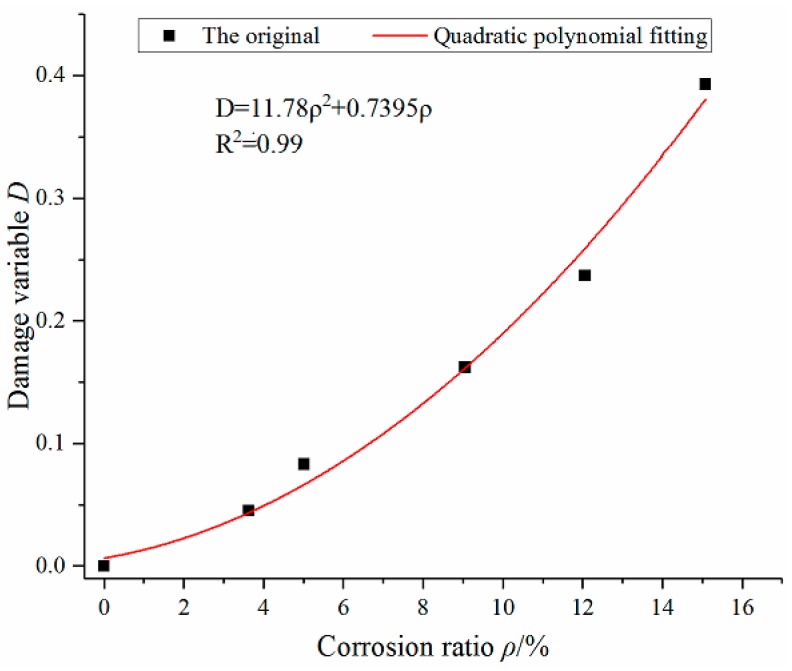
The relationship curve of damage variable *D* and corrosion ratio ρ.

Through the above tests and Equation (25), the damage variable *D* is calculated in Table 5. According to Table 5, the relationship between damage variable *D* and corrosion ratio is drawn (Figure 11), and polynomial curve fitting is performed. Finally, the quadratic polynomial curve is selected, as expressed by Equation (26) and the correlation coefficient reach 0.99.

(26) $$D=11.78\rho^{2}+0.7395\rho$$

### 3.6. Bond-Slip Degradation Constitutive Model Based on Damage Theory

Bond stress *τ* is generally represented by the average value along the bond length, which can be calculated by Equation (27).

(27) $$\tau =\frac{P}{A}$$

where *P* is push-out force, *A* is the surface area of I-shaped steel wrapped by concrete, in this test *A* = 289,200 mm^2^.

#### 3.6.1. Bond-Slip Constitutive Model between Non-Corroded I-Shaped Steel and Concrete

**Figure 12:**
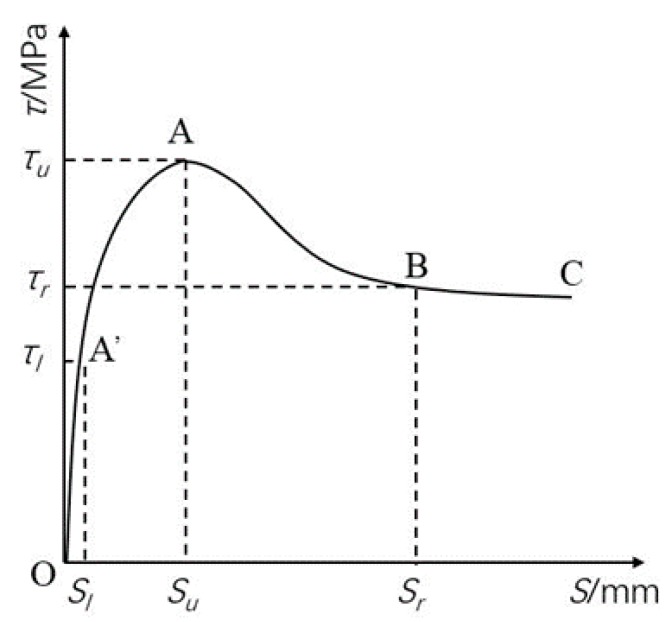
Complete *τ-s* curve and characteristic points.

Based on the research results of many scholars [26,29,35], a complete *τ-s* curve can be obtained, as shown in Figure 12. Three characteristic slip value were defined in *τ-s* curve: ① The turning point slip *S_l_* in the rising segment corresponding to the control point A,(*S_l_,* 0.5*τ_u_*); ② Limit state slip *S_u_*; ③ Initial slip values *S_r_* for residual stage. Due to the limitations of experimental conditions, only the rising segment OA of the τ-s curve was obtained in this experiment, and the descending segment AB and the horizontal residual stage BC were not obtained. Therefore, only the rising segment OA was analyzed in this paper.

The rising segment OA is generally described by hyperbola [35], as expressed by Equation (28).

(28) $$\tau =\frac{S}{aS+b}$$

where $a=\frac{s_{u}-2s_{l}}{\tau_{u}\left(s_{u}-s_{l}\right)}$, $b=\frac{s_{u}s_{l}}{\tau_{u}\left(s_{u}-s_{l}\right)}$.

Through the above data of Figure 8a, the parameters a = 0.102, b = 0.669 of the non-corroded I-shaped steel and concrete can be obtained. The bond-slip constitutive model between non-corroded I-shaped steel and concrete is proposed, as expressed by Equation (29).

(29) $$\tau =\frac{S}{0.102S+0.669}$$

**Figure 13:**
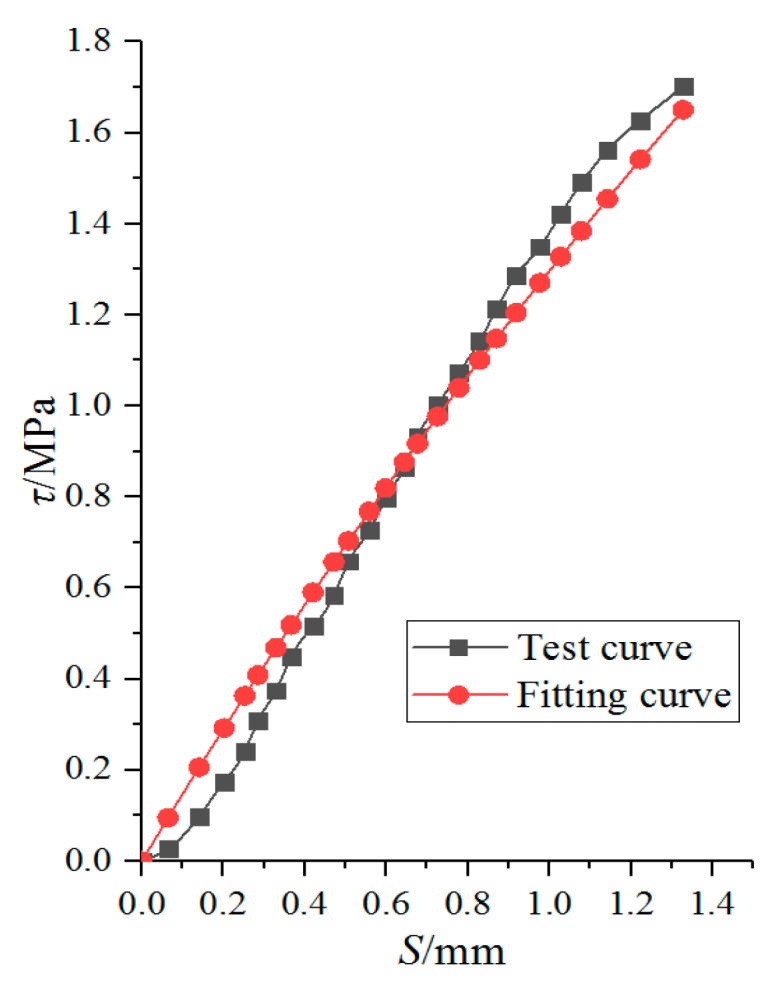
Comparison fitting curve with test curve.

The curve fitted by Equation (29) is compared with the test curve, as shown in Figure 13. It can be seen that the formula fitting curve is in good agreement with the test curve. Therefore, it is feasible to use this formula to express the constitutive relationship of bond-slip between non-corroded I-shaped steel and concrete.

#### 3.6.2. Bond-Slip Degradation Constitutive Model between Corroded I-Shaped Steel and Concrete

Using the constitutive model of bond-slip between non-corroded I-shaped steel and concrete, the degenerate constitutive relationship with damage variable of bond-slip between corroded I-shaped steel and concrete is proposed, as expressed by Equation (30).

(30) $$\tau =(1-D)\frac{S}{0.102S+0.669}=(1-11.78\rho^{2}-0.7395\rho )\frac{S}{0.102S+0.669}$$

Where *τ* is average bond stress, *S* is relative slip, *D* is damage variable, *ρ* is corrosion ratio.

**Figure 14:**
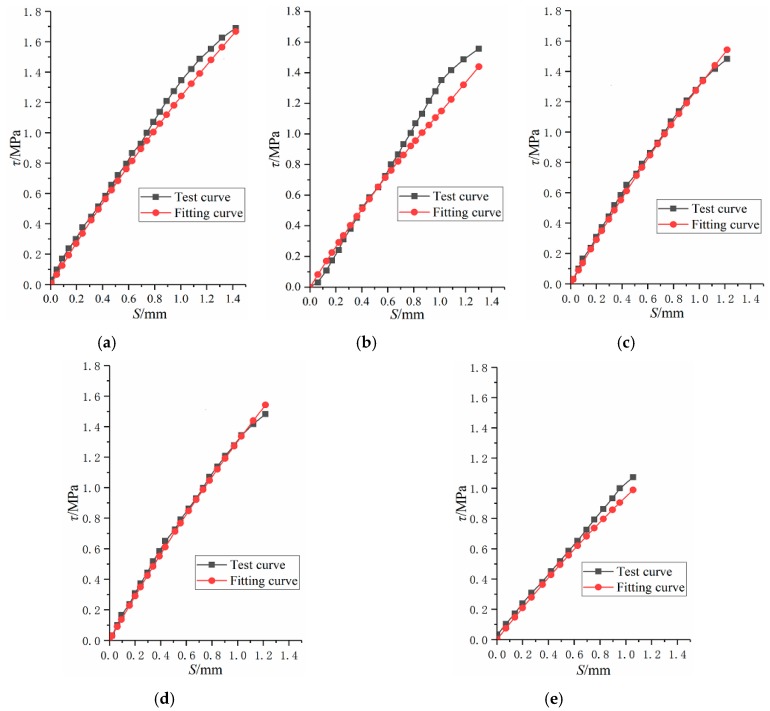
Comparison fitting curve with test curve. (**a**) SRC-2; (**b**) SRC-3; (**c**) SRC-4; (**d**) SRC-5; (**e**) SRC-6.

The curve fitted by Equation (30) is compared with the test curve, as shown in Figure 14. In order to reflect the reliability of the formula, the relative error δ is defined, as expressed by Equation (31). Through data processing and analysis, the range of relative error is about −12%~5%, it indicates that the formula fitting curve is in good agreement with the test curve. Therefore, it is feasible to use this formula to express the bond-slip degradation constitutive model between corroded I-shaped steel and concrete.

(31) $$\delta =\Delta /\tau_{T}=\left(\tau_{F}-\tau_{T}\right)/\tau_{T}\times 100\%$$

where δ is relative error, $\tau_{T}$ is the bond stress obtained by test, $\tau_{F}$ is the bond stress obtained by Equation (30).

## 4. Conclusions

In this paper, experiment programs were carried out to mainly reveal the interfacial bonding characteristics and obtained bond-slip degradation constitutive model between corroded I-shaped steel with different corrosion ratio and concrete, and the following conclusions could be drawn.

1. When the corrosion ratio was less than 15.08%, cracks first appeared in both ends of the flange. With the load increasing, cracks expanded gradually from inside to outside.

2. With the increase of the corrosion ratio, the bond failure load of the specimens presents a decreasing trend. When the corrosion ratio is less than 12%, the failure load decreases linearly. When the corrosion ratio is greater than 12%, the decline rate increases suddenly. Therefore, the corrosion ratio of 12% is a turning point for accelerating degradation of bond-slip performance.

3. The load-slip (*P-S*) curve of the loading end and the free end experienced slip phase and destruction phase in the rising stage.

4. Based on the principle of static equilibrium and minimum potential energy, the distribution value of bond stress along embedment length of I-shaped steel was obtained with a push-out load less than 80% of the bond failure load.

5. Based on the damage theory, the formula for bond-slip degradation constitutive model between corroded I-shaped steel and concrete was established.

## Figures and Tables

**Table 1:** The parameters of I-shaped steel and concrete.

Material Type	Model Specifications	Modulus of Elasticity E (GPa)	Poisson’s Ratio	Density (kg/m^3^)	Yield Strength (MPa)
I-shaped steel	NO.18	210	0.25	7850	235
Concrete	C25	28	0.2	2200	-

**Table 2:** Corresponding relationship between the mass loss and conducting time.

Serial Number	The Original Mass/kg	The Mass after Corrosion/kg	Constant Current/A	The Mass Loss/kg	Conducting Time/h
1	5.79	5.58	5	0.21	48
2	5.713	5.475	5	0.238	60
3	5.763	5.405	5	0.358	89
4	5.723	5.345	5	0.378	97
5	5.95	5.85	5	0.1	20

**Table 3:** Relationship of conduction time and corrosion ratio.

Serial Number	SRC-1	SRC-2	SRC-3	SRC-4	SRC-5	SRC-6
Conduction time/h	0	72	100	180	240	300
Corrosion ratio	0	3.62%	5.02%	9.05%	12.06%	15.08%

**Table 4:** Characteristics of push-out specimens.

Serial Number	Modulus of Elasticity *E*/(GPa)		Cross-Sectional Area A/(cm^2^)		The Perimeter of I-Shaped Steel C_s_/(m)	Embedment Length *l*/(m)
	*E_c_*	*E_s_*	*A_c_*	*A_s_*		
SRC-1~6	28	210	1569.4	30.6	0.72	0.4

**Table 5:** Damage variable *D* under different corrosion ratio.

Serial Number	Corrosion Ratio *ρ*/(%)	Bond Failure Load *P_u_*/kN	Damage Variable *D*
SRC-1	0	511	0
SRC-2	3.62	488	0.045
SRC-3	5.02	468	0.084
SRC-4	9.05	428	0.162
SRC-5	12.06	390	0.237
SRC-6	15.08	310	0.393

## References

[1] Dammyr O., Nilsen B., Gollegger J. (2017). Feasibility of tunnel boring through weakness zones in deep Norwegian subsea tunnels. Tunn. Undergr. Space Technol..

[2] Wang M. (2008). Current developments and technical issues of underwater traffic tunnel—Discussion on construction scheme of Taiwan strait undersea railway tunnel. Chin. J. Rock Mech. Eng..

[3] Liao C., Guo X. (2008). Key technologies in subsea tunnel construction in China. Tunn. Constr..

[4] Qing S., Xie W., Gao W., Huang S. (2011). Innovation of key construction technology for Kiaochow Bay Submarine Tunnel with boring and blast method. J. Railw. Eng. Soc..

[5] Chiorean C.G., Buru S.M. (2017). Practical nonlinear inelastic analysis method of composite steel-concrete beams with partial composite action. Eng. Struct..

[6] Lacki P., Nawrot J., Derlatka A., Winowiecka J. (2019). Numerical and experimental tests of steel-concrete composite beam with the connector made of top-hat profile. Compos. Struct..

[7] Thomas A. (2008). Sprayed Concrete Lined Tunnels.

[8] Wang J.B., Niu D.T., Zhang Y.L. (2015). Mechanical properties, permeability and durability of accelerated shotcrete. Constr. Build. Mater..

[9] Hou D.S., Li T., Wang P. (2018). Molecular Dynamics Study on the Structure and Dynamics of NaCl Solution Transport in the Nanometer Channel of CASH Gel. ACS Sustain. Chem. Eng..

[10] Shi X.M., Xie N., Fortune K., Gong J. (2012). Durability of steel reinforced concrete in chloride environments: An overview. Constr. Build. Mater..

[11] Revie R.W. (2008). Corrosion and Corrosion Control: An Introduction to Corrosion Science and Engineering.

[12] Böhni H. (2005). Corrosion in Reinforced Concrete Structures.

[13] Hou L.J., Guo S., Zhou B.X., Chen D., Aslani F. (2019). Bond-slip behaviour of corroded reinforcement and ultra-high toughness cementitious composite in flexural members. Constr. Build. Mater..

[14] Lin H.W., Zhao Y.X., Feng P., Ye H.L., Ozbolt J., Jiang C., Yang J.Q. (2019). State-of-the-art review on the bond properties of corroded reinforcing steel bar. Constr. Build. Mater..

[15] Zhao W.P., Zhu B.R. (2018). Theoretical model for the bond-slip relationship between ribbed steel bars and confined concrete. Struct. Concr..

[16] Jiang C., Wu Y.F., Dai M.J. (2018). Degradation of steel-to-concrete bond due to corrosion. Constr. Build. Mater..

[17] Hou L.J., Zhou B.X., Guo S., Zhuang N., Chen D. (2018). Bond-slip behavior between pre-corroded rebar and steel fiber reinforced concrete. Constr. Build. Mater..

[18] Wang J., Yin R., Zhu Y., Chen J., Zhang S. (2018). Experimental Study on Corroded Steel Ban with the Center of the Recycled Concrete Bond Slip Out. Bull. Chin. Ceram. Soc..

[19] Hou L.J., Liu H., Xu S.L., Zhuang N., Chen D. (2017). Effect of corrosion on bond behaviors of rebar embedded in ultra-high toughness cementitious composite. Constr. Build. Mater..

[20] Lin H.W., Zhao Y.X., Ozbolt J., Hans-Wolf R. (2017). The bond behavior between concrete and corroded steel bar under repeated loading. Eng. Struct..

[21] Ma Y.F., Guo Z.Z., Wang L., Zhang J.R. (2017). Experimental investigation of corrosion effect on bond behavior between reinforcing bar and concrete. Constr. Build. Mater..

[22] Feng Q., Visintin P., Oehlers D.J. (2016). Deterioration of bond-slip due to corrosion of steel reinforcement in reinforced concrete. Mag. Concr. Res..

[23] Bryson J.O., Mathey R.G., Hunaiti Y.M. (1962). Surface condition effect on bond strength of steel beams in concrete. J. ACI.

[24] Roeder C.W. (1985). Bond Stress of Embedded Steel Shapes in Concrete, Composite and Mixed Construction.

[25] Hamdan M., Hunaiti Y. Factors affecting bond strength in composite columns. Proceedings of the 3rd International Conference on Steel-Concrete Composite Structures.

[26] Yang Y., Guo Z., Xue J., Zhao H., Nie J. (2005). Experiment study on bond slip behavior between section steel and concrete in SRC structures. J. Build. Struct..

[27] Shansuo Z., Guozhuan D., Yong Y., Maohong Y., Junfeng Z. (2003). Experimental study of bond-slip performance between steel and concrete in SRC structures. Eng. Mech..

[28] Zheng H., Hu X., Liu J., Wan H. (2015). Experimental study on bond slip behavior between section steel and concrete in partially encased composite members. Ind. Constr..

[29] Liu C., Lv Z., Bai G., Yin Y. (2018). Experiment study on bond slip behavior between section steel and RAC in SRRC structures. Constr. Build. Mater..

[30] Chen L.H., Wang S.Y., Yin C., Li S.T. (2019). Experimental study on constitutive relationship between checkered steel and concrete. Constr. Build. Mater..

[31] Wang Y., Wang C., Zhu D., Xu D., Fu C. (2016). Study on Performance of Bond-slip between Steel Shapes and Concrete under High Temperatures. J. Disaster Prev. Mitig. Eng..

[32] Sokolnikoff I.S. (1983). Mathematical Theory of Elasticity.

[33] Roeder C.W., Chmielowski R., Brown C.B. (1999). Shear connector requirements for embedded steel sections. J. Struct. Eng..

[34] Hu H. (1984). Variational Principles of Theory of Elasticity with Applications.

[35] Yong Y. (2003). Study on the basic theory and its application of bond-slip between steel shape and concrete in SRC structures. Xi’an Univ. Archit. Technol..

[36] Li J., Wu J. (2005). Elastoplastic damage constitutive model for concrete based on damage energy release rates, part I: Basic formulations. China Civil Eng. J..

[37] Wu J., Li J. (2005). Elastoplastic damage constitutive model for concrete based on damage energy release rates, part II: Numerical algorithm and verifications. China Civil Eng. J..

